# Effects of Sphingosine-1-Phosphate on the Facilitation of Peripheral Nerve Regeneration

**DOI:** 10.7759/cureus.73784

**Published:** 2024-11-15

**Authors:** Chi Li, Toru Yamamoto, Hiroko Kanemaru, Naotaka Kishimoto, Kenji Seo

**Affiliations:** 1 Division of Dental Anesthesiology, Faculty of Dentistry & Graduate School of Medical and Dental Sciences, Niigata University, Niigata, JPN; 2 Division of Oral Management Clinic for Medical Cooperation, Niigata University Medical and Dental Hospital, Niigata, JPN

**Keywords:** fingolimod, fty720, peripheral nerve regeneration, s1p1 receptor, schwan cell, sphingosine-1-phosphate

## Abstract

This study aims to explore the role of sphingosine-1-phosphate (S1P) in peripheral nerve regeneration after injury. S1P is a crucial metabolite involved in cell migration, inflammation, and nerve regeneration. In this research, six-week-old male Sprague-Dawley rats (total n=18) underwent transection of the inferior alveolar nerve (IAN) and were divided into three groups: S1PR agonist (FTY720) (n=6), saline control (n=6), and S1P1R antagonist (n=6). Regeneration was assessed using immunostaining and retrograde tracing. Results showed that the S1PR agonist group had superior axonal and Schwann cell regeneration compared to controls. Additionally, the combination with S1P1R antagonists inhibited the effects of the agonists, further confirming the potential role of S1P1R in nerve repair. Our results suggest that mediating S1P1R signaling could facilitate the regeneration of peripheral nerves after injury.

## Introduction

Dental treatments and oral surgeries involving implants can sometimes damage the inferior alveolar nerve (IAN), significantly reducing the patient's quality of life (QOL) due to resulting perceived dysfunction and persistent pain. Clinically, interventions such as vitamin supplementation, physiotherapy, and satellite ganglion block (SGB) are currently being performed. However, there is a need to elucidate the optimal mechanism for peripheral nerve regeneration.

Sphingolipids, key components of cell membranes, are built around the amino alcohol sphingosine. One of their metabolic products, sphingosine-1-phosphate (S1P), is stored within cells and can be released to act extracellularly. S1P binds to its receptors, known as S1P receptors (S1PRs), which are involved in various signaling pathways that affect critical cellular processes [1,2]. As S1PRs couple to a variety of G proteins, stimulation of S1PRs can impact several signal transduction pathways and cellular processes.

Schwann cells are essential neuroglial cells in the peripheral nervous system, and their function is important for the normal functioning of neurons. These cells possess multi-layered lipid membranes containing myelin proteins, which facilitate rapid electrical signal transmission. After peripheral nerve injury, Schwann cells dedifferentiate, assist in myelin clearance, attract macrophages for debris removal, and support axonal regeneration. They re-differentiate to re-enwrap regenerated axons, forming new myelin sheaths [3]. Schwann cells express S1PR, which influences their migration and cytoskeletal dynamics, and interferes with myelin formation in vitro [4].

Clinically, the S1PR ligand fingolimod (FTY720) is used to treat relapsing-remitting multiple sclerosis because of its immunomodulatory properties. It is neuroprotective in various mouse disease models [5]. Recent data suggest that its activation also has axon-protective properties [6], as well as the promotive effect of regeneration in the optic and facial nerve [7,8]. However, it remains unclear which subtype of S1PR activates peripheral nerve regeneration. The aim of this study was to investigate the potential therapeutic effects of S1P1R modulation on peripheral nerve regeneration after injury and to accumulate relevant therapeutic evidence after dental surgery-related nerve damage.

## Materials and methods

Animals

Eighteen male Sprague-Dawley rats (6 weeks old, 180-220 g) were used. All procedures were approved by the Niigata University Intramural Animal Use and Care Committee (Approval Number: SA00146) and conducted according to their guidelines. Rats were housed under temperature (25°C) and humidity (approximately 40%) controlled conditions, with a 12-h light/dark cycle and free access to food and water. Animals were assigned to experimental groups using computer-generated random numbers and the allocation table.

Inferior alveolar nerve transection

**Figure 1:**
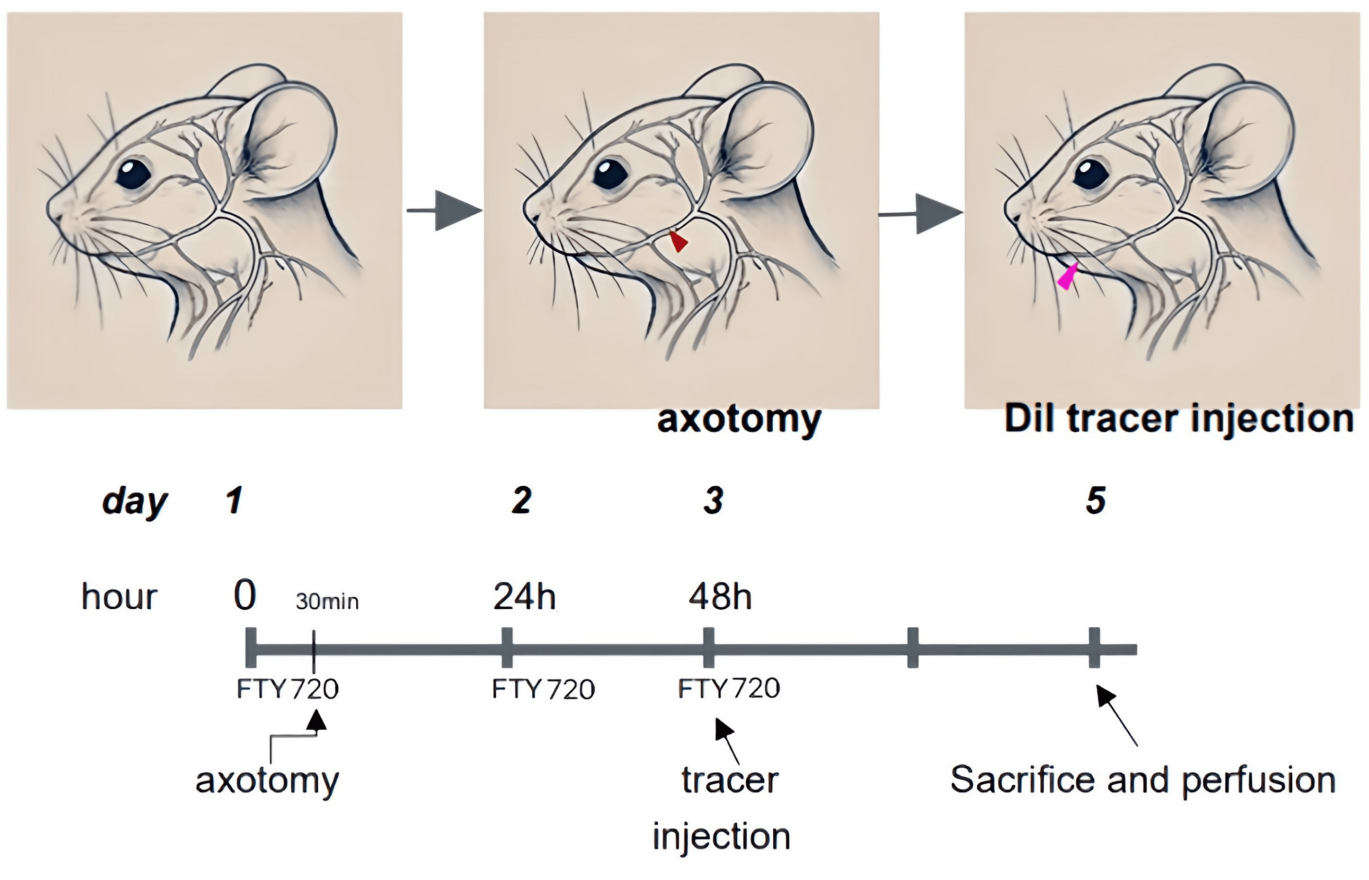
Experimental outline of the inferior alveolar nerve injury model

Rats were anesthetized and maintained at a constant body temperature using a heating plate. The hair on the left cheek was removed, and the skin was sterilized. A small incision was made to expose the inferior alveolar nerve (IAN), which was transected by 1 mm, and the incision was closed with non-absorbable sutures (Figure 1). 

Administration of S1PR agonist and antagonist

In FTY720 group rats (n=6), FTY720 was administered intraperitoneally at 1 mg/kg daily for 3 days post-surgery. Control group rats (n=6) received saline injections for 3 days post-surgery. In the antagonist group, rats (n=6) were administered 1 mg/kg NIBR-0213 (S1P1R antagonist) together with 1 mg/kg FTY720 every day for 3 days post-surgery (Figure 1). Analgesics were provided in all groups for 3 days post-surgery.

Neuronal tracer infusion

The DiI neuronal tracer can be injected into neurons where it forms a stable staining pattern on the cell membrane, allowing visualization of the neuronal morphology, synaptic connections, and neural circuits. DiI tracer was injected into the submandibular region of the rat's cheek using a syringe. After 2 days, rats were perfusion-fixed, and the trigeminal ganglions (TGs) were processed for sectioning and fluorescent staining (Figure 1). 

Tissue processing

Rats were deeply anesthetized with a combination of anesthetics (2.5 mg/kg butorphanol, 0.375 mg/kg medetomidine, and 2 mg/kg midazolam, i.p.) and transcardially perfused with phosphate-buffered saline (PBS) and 4% paraformaldehyde. Sections for immunohistochemistry of the saline, FTY720, and FTY720+NIBR-0213 (S1P1R antagonist) groups were obtained from specimens on day 5 after surgery.

The surgical side of the mandible, including the IAN and the TG, was excised. The specimens were cut into 25 µm slices for fluorescence staining using a cryostat (CM1850, Leica Biosystems, Germany).

Immunohistochemistry

Sections were incubated with primary antibodies against β3-tubulin for axons of neurons (1:500 dilution, ab52623, Abcam, UK) and S100β for Schwann cells (1:500 dilution, ab52642, Abcam, UK) for 24 hours at 4°C. This was followed by incubation with the secondary antibodies (1:500 dilution; Alexa Fluor 488, ab150081) for 1 hour at room temperature. The dilution concentration was determined by preliminary experiments within the manufacturer's recommended dilution range (1:50 ~ 1:500) to obtain the optimal image with minimal non-specific reactivity [9]. As an antibody specificity and validation step, we confirmed no positive immunoreactivity for the antibody targets in tissue sections incubated without the primary antibody in the preliminary experiment. Images of slides were captured using a fluorescence microscope (BZ-X800, Keyence, Tokyo, Japan). The various image capture settings were kept consistent across all groups. The middle section of the IAN and all the sections of the TG were used to quantify the % area of regenerated nerve to missing area in the IAN and the number of DiI-positive neurons in the TG (3rd branch region for the IAN) using ImageJ software (National Institutes of Health, Bethesda, MA, USA). The number of DiI-positive neurons in the TG was counted, and the average of the three sections was used as the representative value for the individual rat.

Statistical analysis

Statistical comparisons were made using GraphPad Prism 9 (GraphPad, CA, USA). Unpaired t-tests and one-way analyses of variance (ANOVA) were used. Statistical significance was set at p<0.05. All data were expressed as mean values±standard error of the mean (SEM). 

## Results

Effects of S1PR agonist on nerve regeneration

**Figure 2:**
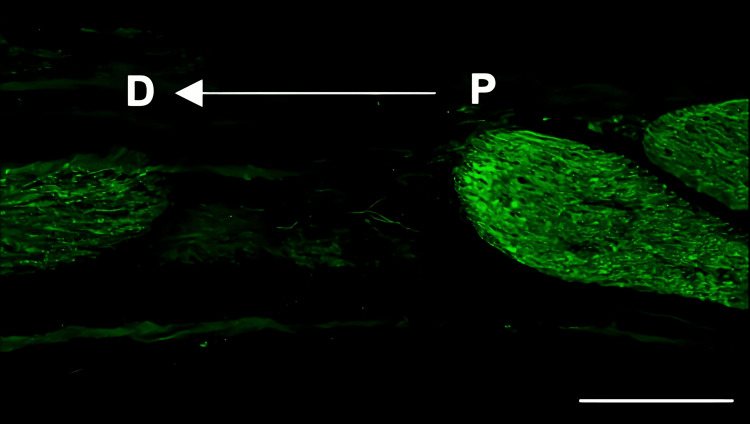
Immunohistochemistry of S100β in the inferior alveolar nerve from the saline group Scale bar = 200 µm. P: proximal side, D: distal side

**Figure 3:**
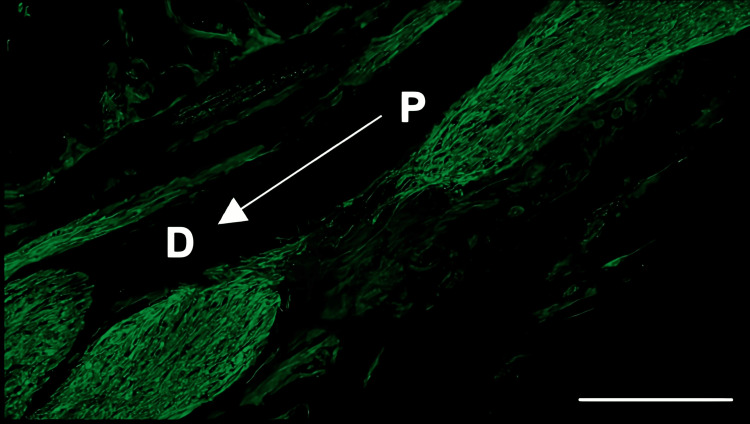
Immunohistochemistry of S100β in the inferior alveolar nerve from the FTY720 group Scale bar = 200 µm. P: proximal side, D: distal side

**Figure 4:**
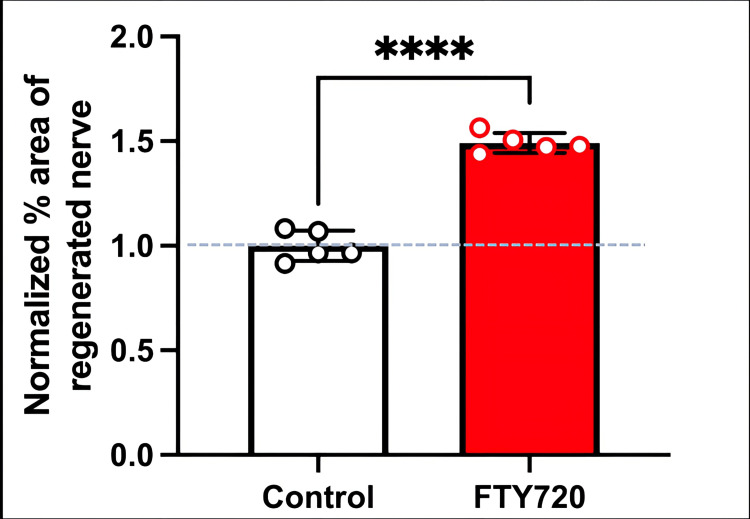
Normalized % area of the regenerated nerve stained with S100β in the control (saline) and FTY720 groups Note that a higher percentage area of regenerated nerves was observed in FTY720-treated rats compared to control (saline) rats (****P<0.0001, unpaired t-test, n=5, respectively).

**Figure 5:**
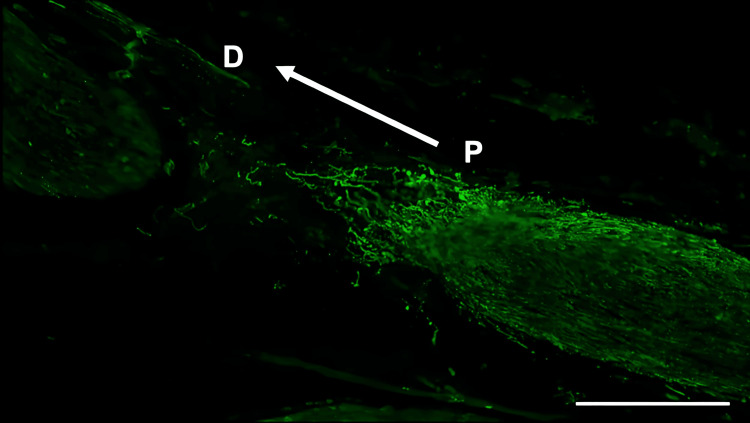
Immunohistochemistry of β3-tubulin in the inferior alveolar nerve from the saline group Scale bar = 200 µm. P: proximal side, D: distal side

**Figure 6:**
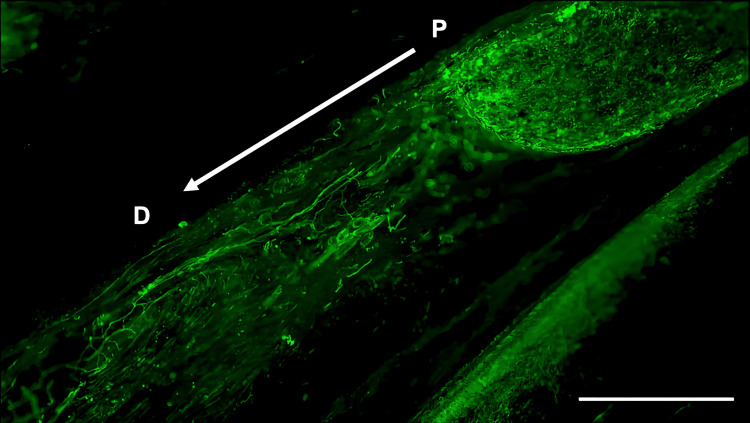
Immunohistochemistry of β3-tubulin in the inferior alveolar nerve from the FTY720 group Scale bar = 200 µm. P: proximal side, D: distal side

**Figure 7:**
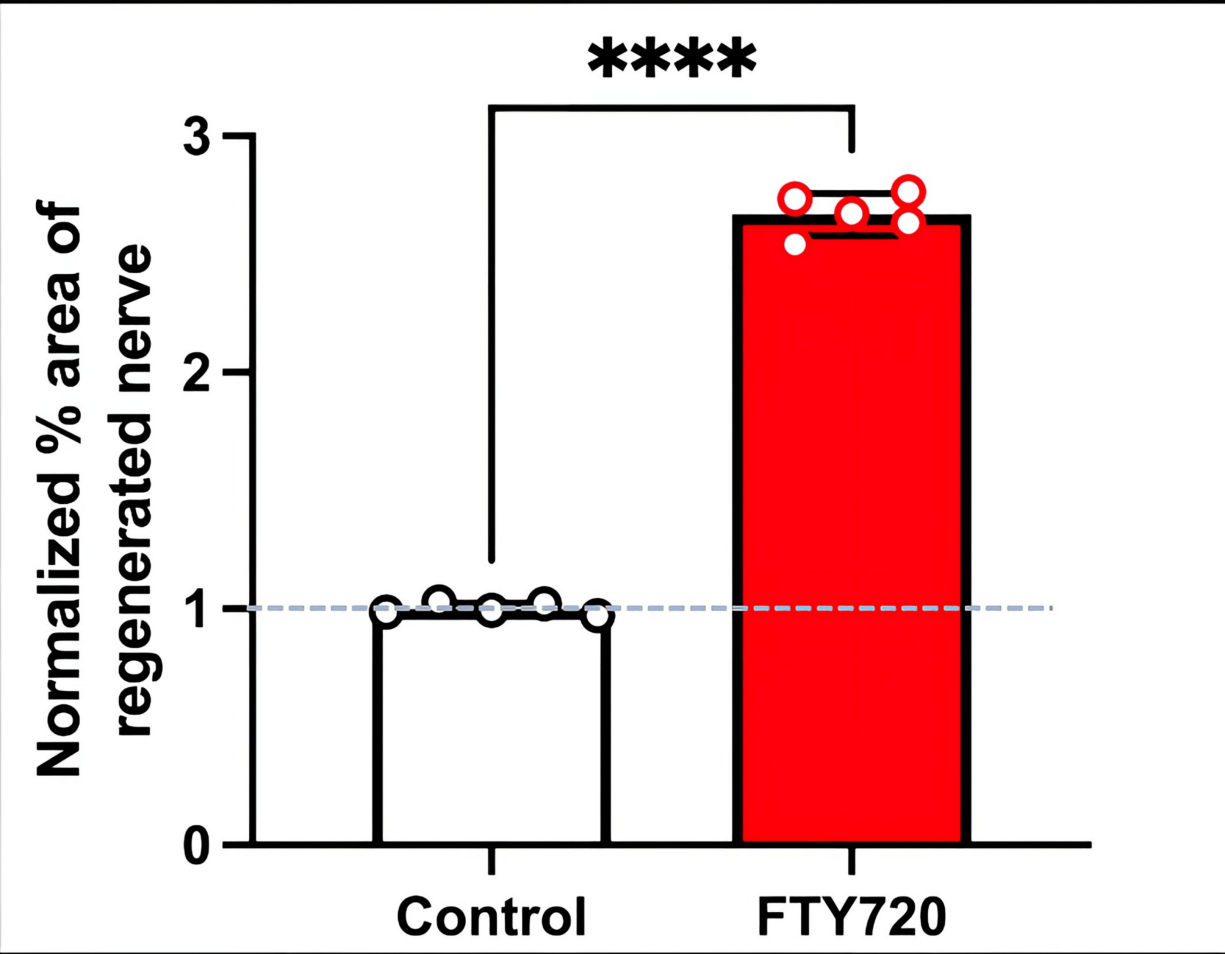
Normalized % area of regenerated nerves stained with β3-tubulin in the control (saline) and FTY720 groups Note that a higher percentage area of regenerated nerve was observed in FTY720-treated rats compared to control (saline) rats (****P<0.0001, unpaired t-test, n=5, respectively).

Histological analysis of the inferior alveolar nerve from the proximal (P) to distal (D) end on day 5 post-injury revealed that Schwann cell and axonal regeneration was enhanced in the FTY720-treated group compared with controls (Figures 2-7), showing more pronounced axonal regeneration and nerve network reconstruction following treatment.

Regeneration of damaged axons by S1PR activation

**Figure 8:**
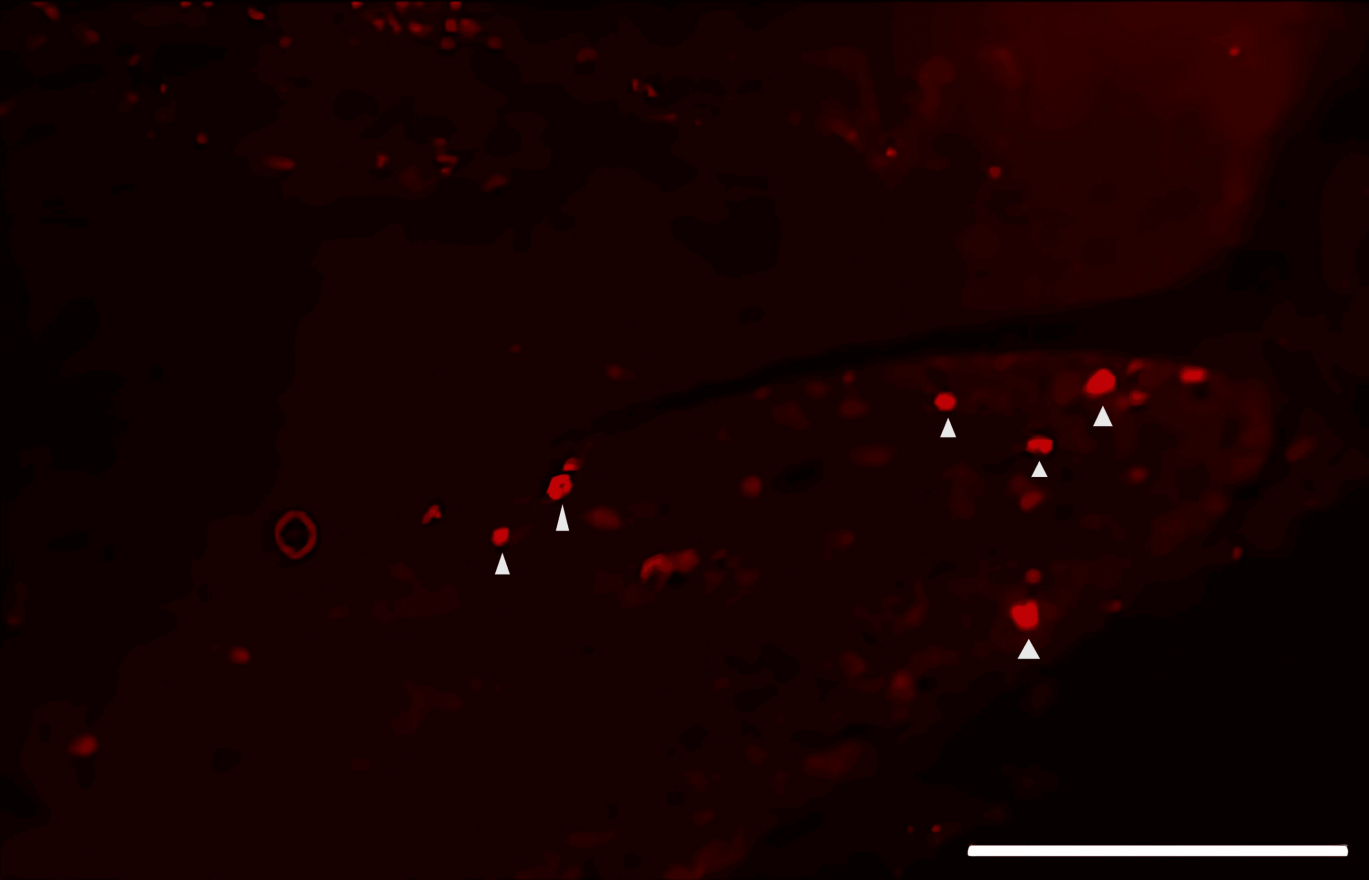
Fluorescence image of neuronal tracer DiI-positive neurons in the trigeminal ganglion from the saline group Triangle mark: DiI-positive neuron. Scale bar = 100 µm.

**Figure 9:**
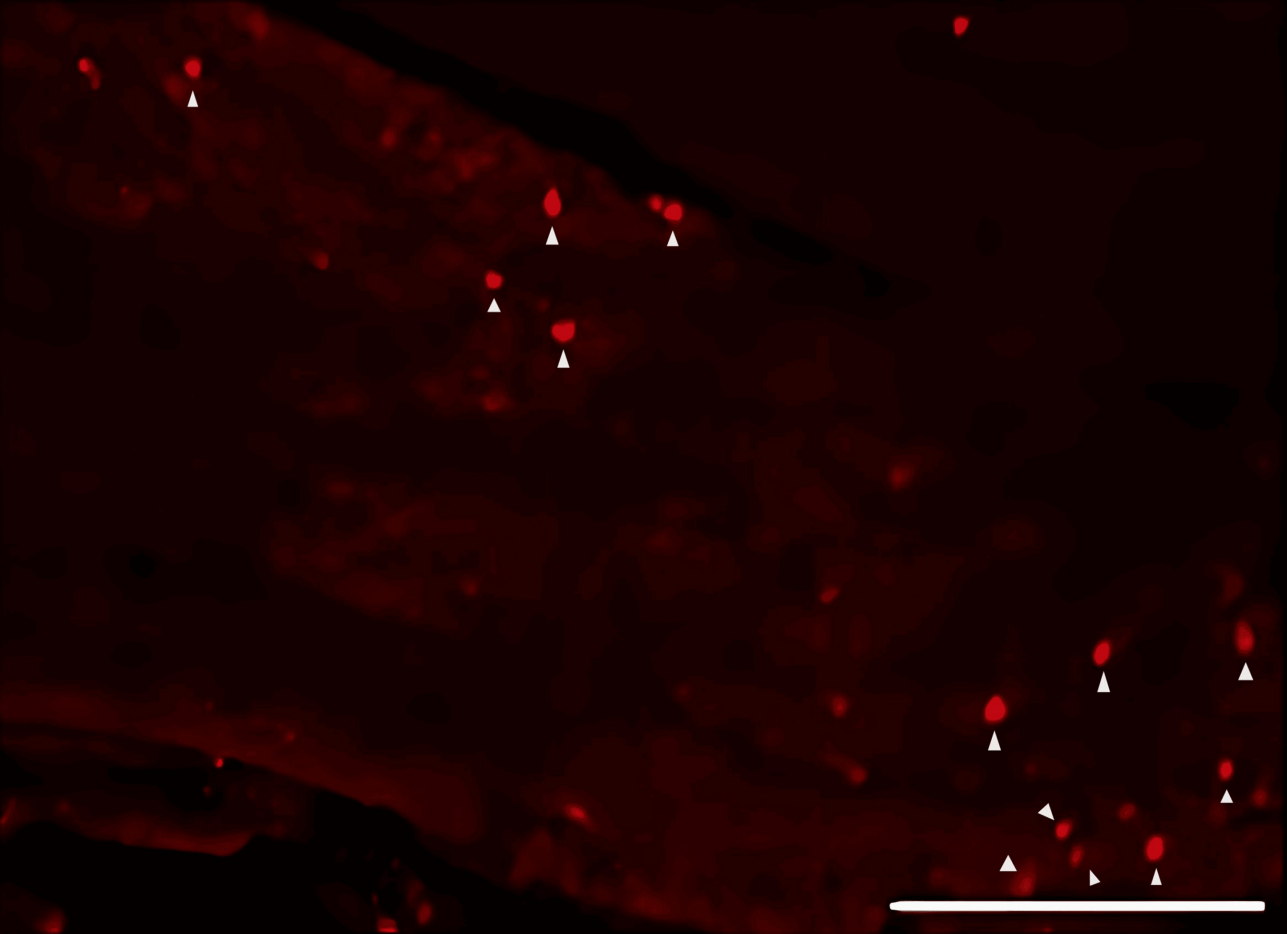
Fluorescence image of neuronal tracer DiI-positive neurons in the trigeminal ganglion from the FTY720 group Triangle mark: DiI-positive neuron. Scale bar = 100 µm.

**Figure 10:**
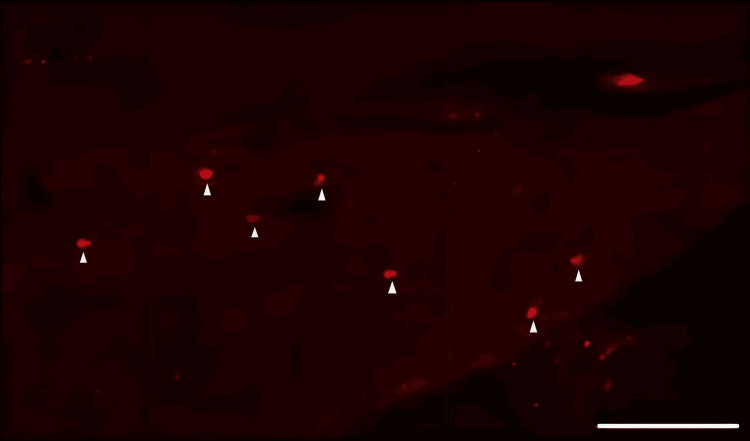
Fluorescence image of neuronal tracer DiI-positive neurons in the trigeminal ganglion from the NIBR-0213 group Triangle mark: DiI-positive neuron. Scale bar = 100 µm.

**Figure 11:**
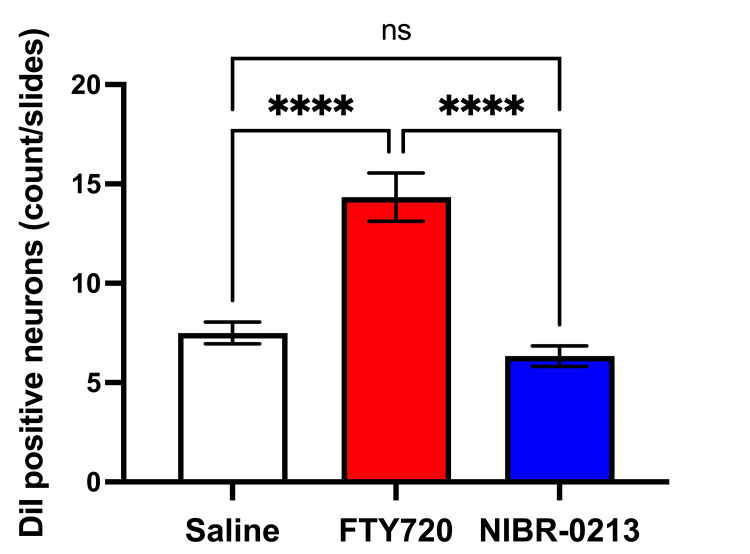
Quantification of Dil-positive neurons in the TG among the three treatment groups. ****P<0.0001, one-way ANOVA with Šídák's test was used for multiple comparisons in the saline group vs. FTY720 group and in the FTY720 group vs. FTY720 + NIBR-0213 group, n=5, respectively.

DiI tracing showed higher numbers of DiI-positive cells in the TG of the experimental group of rats treated with FTY720 when compared with the controls (Figure 8, 9). Co-administration of the S1P1R antagonist NIBR-0213 diminished these effects (Figure 10), highlighting the involvement of S1P1R in facilitating peripheral nerve regeneration (Figure 11).

## Discussion

The multiple sclerosis (MS) drug, fingolimod (FTY720) has been shown to have beneficial effects on central nervous system (CNS) remyelination [7,9,10]. MS is a type of autoimmune neurological disease in which the myelin sheath that surrounds nerve fibers in the nervous system, such as the brain, spinal cord, and optic nerves, becomes inflamed, damaging (demyelinating) the myelin that covers the axons of nerve cells (neurons) [11].

In the optic nerve, which is part of the central nervous system, fingolimod (FTY720) has been reported to improve functional recovery and myelin preservation of the optic nerve pathway in a localized demyelination model of the rat optic chiasm [7]. The underlying mechanism is thought to be that FTY720 stimulates neuronal gene expression, axonal growth, and regeneration [8].

Also, in the peripheral nerve, accumulating evidence of fingolimod (FTY720) in the dorsal root ganglion (DRG) region has been reported. Additionally, FTY720 has been reported to promote nerve regeneration and recovery of motor function in a rat model of spinal cord injury [12,13]. Moreover, after spinal cord injury, fibrous scarring inhibits axonal regeneration and hinders recovery of neural function. However, FTY720 has been reported to suppress fibrous scar formation by inhibiting T cell-derived IFN-γ and contribute to neurological recovery after spinal cord injury [14].

Therefore, in this study, we aimed to investigate the effect of the S1PR agonist FTY720 on peripheral nerve regeneration after trigeminal nerve injury using IAN as one of the branches of trigeminal nerves. With regard to the evaluation of peripheral nerve regeneration at the injured site of the IAN, the FTY720 group showed a higher % area of regenerated nerve compared to the saline group and FTY720+NIBR-0213 group, suggesting the involvement of FTY720 in the promotion of peripheral nerve regeneration. Regarding the results of DiI neuronal tracer from peripheral IAN to neuron somata (cell body) in TG, the number of DiI-positive neurons in the TG was significantly increased in the FTY720 group compared to the saline group, and similar to the saline group in the antagonist group. DiI was used as a neuronal tracer to retrogradely label neuronal cell bodies in the TG. The presence of DiI-positive neurons is thought to reflect the state of reconnection of severed nerve axons, suggesting that these data support the nerve regeneration-promoting effect of FTY720.

Our results demonstrated that the agonism of the S1PR with FTY720 significantly enhances nerve regeneration by increasing Schwann cell and axonal regeneration. Additional antagonism of S1P1Rs with NIBR-0213 reduced these effects, suggesting that specific S1P1R signaling is involved in promoting nerve repair after injury. These findings align with those of previous studies, which suggest that S1P signaling regulates cellular processes essential for nerve regeneration, including Schwann cell differentiation and the release of neurotrophic factors. Schwann cells are pivotal in the peripheral nervous system for their role in myelination and support of axonal growth [3]. Therefore, S1PR activation appears to create a favorable milieu for axonal sprouting and regeneration. The effect of FTY720 may involve internalization of the receptor and modulation of signaling pathways [15]. Regarding the signaling pathway, another study indicated that fingolimod promotes peripheral nerve regeneration via modulation of lysophospholipid signaling [16].

In addition to axon growth evaluated by β3-tubulin (Figures 5-7), we observed more Schwann cells in injured inferior alveolar nerves in the FTY720 treatment group compared with the control (saline) group (Figures 2-4). This is consistent with previous reports suggesting that FTY720 may stimulate a Schwann cell phenotype that promotes regeneration [17]. On the other hand, there is a report that FTY720 does not promote nerve regeneration [18], but this may be due to differences in experimental models. It was not clear which of the five S1P receptors originally expressed on Schwann cells are responsible for fingolimod's promotion of cellular dedifferentiation [19]. In Dil tracing at the TG, compared with the control group, the experimental group treated with an S1P receptor agonist FTY720 showed a significant increase in the number of Dil-positive cells, while the group treated simultaneously with an S1P receptor agonist and blocker exhibited a marked decrease in Dil-positive cell count. Therefore, our results suggest at least partial involvement of S1P1R in facilitating peripheral nerve regeneration.

There are some limitations in the present study. First, in the present study, of the S1P1-5 subtypes, only S1P1 was examined. Further research is needed to determine whether there are interactions with other subtypes. Second, as drug dose and route variability, the study uses fixed doses of fingolimod (FTY720) without exploring a dose-response relationship or varying administration routes. This limits the understanding of the efficacy and safety of these treatments in different scenarios. Future studies should investigate the optimal administration timing and dosage of FTY720. Third, lack of long-term follow-up: the study focuses on the effects of treatments in the acute phases (5 days after nerve injury) but does not assess long-term outcomes. Extending the observation period would address this gap to evaluate the sustainability of the regenerative effects. Schwann cell reprogramming and differentiation are critical for neuronal regeneration and remyelination following a peripheral nerve injury. The p75 neurotrophin receptor has been identified as a positive modulator of Schwann cell myelination during development and has been implicated in promoting nerve regeneration after injury [20-22]. Moreover, nerve growth factor (NGF) plays an important role in reconstructing peripheral nerve fibers [23]. We also should have examined the change in expression of the p75 neurotrophin receptor and NGF.

FTY720 is an effective treatment; however, it can cause side effects such as bradycardia [24] due to FTY720 affecting multiple S1PR subtypes (S1P2~5) in addition to S1P1R. However, the S1P signaling pathway, particularly through the S1P1R, holds promise as a therapeutic strategy to enhance peripheral nerve regeneration. Therefore, developing a selective S1P1R agonist is crucial for avoiding complications in treating nerve injuries. Future research should refine experimental designs, investigate mechanisms, and conduct translational studies to advance from preclinical results to clinical practice. Further research in this area is essential, including exploring dose-response effects or investigating other S1P receptor subtypes in nerve regeneration.

## Conclusions

This study provides insights into the role of S1PR in peripheral nerve regeneration after injury. Future research is needed to explore further mechanisms and to evaluate the clinical applicability of S1P1R agonists in nerve repair and regeneration.
